# Hemodynamic behavior of stentless aortic valves in long term follow-up

**DOI:** 10.1186/s13019-014-0197-2

**Published:** 2014-12-20

**Authors:** Torsten Christ, Herko Grubitzsch, Benjamin Claus, Georg Heinze, Simon Dushe, Wolfgang Konertz

**Affiliations:** Department of Cardiovascular Surgery, Charité-Universitätsmedizin Berlin, Charitéplatz 1, Berlin, 10117 Germany

## Abstract

**Objectives:**

Stentless aortic valve replacements show improved hemodynamics due to larger orifice area and lower transvalvular gradients in short and mid-term follow-up. Hemodynamic long-term behavior and the adaptation of the left ventricle as well as valve-durability in patients aged ≤60 years remains unclear.

**Methods:**

7 to 16 years after aortic valve replacement, 54 patients (mean age at operation 53.1 ± years) received echocardiography and clinical examination. Mean follow-up time was 10.8 ± 2.2 years. Evaluated were NYHA class, transvalvular gradients, estimated aortic valve orifice area, degree of aortic valve insufficiency, left ventricular mass and function.

**Results:**

At follow-up only one patient presented with NYHA class III. All other patients were in NYHA class I or II. Maximum and mean pressure gradients of the prostheses were 16.3 ± 7.4 mmHg and 9.1 ± 4.2 mmHg, respectively. Compared to echocardiography at discharge the mean pressure gradients dropped 18.0% (2.0 ± 0.9 mmHg) and stayed stable until 14 years after the operation. Only 5 patients showed relevant regurgitation (at 13–16 years after valve replacement), 49 showed no or trivial regurgitation. Left ventricular mass had decreased 26.5% (107.9 ± 18.5 g). Left ventricular ejection fraction (LVEF) had increased in most patients and decreased in only one. For patients with preoperatively impaired left ventricular function an increase of LVEF of 13.1 ± 3.1% was seen.

**Conclusion:**

Porcine stentless aortic valves provide excellent hemodynamic long-term results without significant rise of transvalvular pressure gradients or relevant insufficiencies until 14 years after implantation, leading to sustained decrease of left ventricular mass and improvement of left ventricular function.

**Electronic supplementary material:**

The online version of this article (doi:10.1186/s13019-014-0197-2) contains supplementary material, which is available to authorized users.

## Background

Stentless aortic valve replacements (SAVR) were designed by avoiding an obstructive stent [1]. Due to this difference, SAVR show a larger orifice area, lower transvalvular gradients and improved hemodynamics compared to stented aortic valve replacement [2]. The occurrence of a patient-prosthesis-mismatch could be ruled out with SAVR [3]. These advantages led in comparison to stented bioprostheses to reduced left ventricular mass and improved survival in short and midterm follow-up [4]. It may also lead to improved survival and left ventricular function in long-term follow-up. The main disadvantage of bioprostheses is their limited durability, especially in younger patients. Therefore the American Heart Association and the American College of Cardiology recommend the use of bioprosthetic aortic valve replacements in patients above the age of 60 years, for patients under the age of 60 years the use of mechanical aortic valve replacements is recommended [5]. Nonetheless, in long term follow-up survival between mechanical and stented bioprostheses slightly favored the latter [6], despite the higher risk of reoperation. The superior hemodynamic properties of SAVR may lead to a higher durability compared to stented bioprostheses. We already reported intermediate to long term results of SAVR in patients under 60 years [7],[8]. Yet no long-term follow-up of the hemodynamic performance of SAVR with its implication on durability in younger patients has been published.

## Methods

The study was approved by the Ethikausschuss institutional review board on the local Ethics Committee.

We identified patients who had received a porcine SAVR in our center 7 to 16 years ago and were 60 years old or younger at the time of operation. Of the identified 64 patients with the originally implanted valve still in place 54 patients gave informed consent to the clinical trial and received clinical examination and echocardiography. Mean age of the patients at operation was 53.1 ± 7.7 years. Mean follow-up time was 10.8 ± 2.2 years. Captured and analyzed were NYHA class, maximum and mean transvalvular pressure gradient, effective aortic valve orifice area (EOA), degree of aortic valve insufficiency, left ventricular mass and left ventricular function. Standard techniques were used to obtain echocardiographic measurements, in accordance with the guidelines of the German Society of Ultrasound in Medicine [9]. Pulsed wave Doppler was used to measure mean and maximum systolic blood flow velocities in the left ventricular outflow tract, and continuous wave Doppler was used to measure systolic blood flow velocities across the aortic valve. Peak and mean transvalvular gradients were obtained using the modified Bernoulli equation. The EOA and left ventricular mass were calculated with the continuity equation [10] and the ASE equation [11], respectively.

### Statistical analysis

All data were analyzed with IBM SPSS Statistics version 21 (IBM Corporation). Descriptive statistics are reported as the mean ± standard deviation for continuous variables and as frequencies and percentages for categorical variables, unless otherwise noted. Normal distribution was tested by the Shapiro-Wilk-test. Normally distributed data were analyzed using the Students *t*-test, while the Wilcoxon test was used for non-normally distributed data. All p values were two-sided. Statistical significance was set at a p value of less than 0.05.

## Results

Five different porcine SAVR were implanted in the study population. Implanted valves were: 36 Edwards Prima Plus, 12 St. Jude Medical Toronto SPV™, 3 Vascutek Elan™, 2 Medtronic Freestyle® and 1 Shelhigh stentless bioprosthesis. Implanted valves sizes varied between 25 mm and 29 mm in diameter. Mean valve size was 27.4 ± 1.3 mm.

At follow-up only one patient presented with NYHA class III. All other patients were in NYHA class I or II.

**Figure 1:**
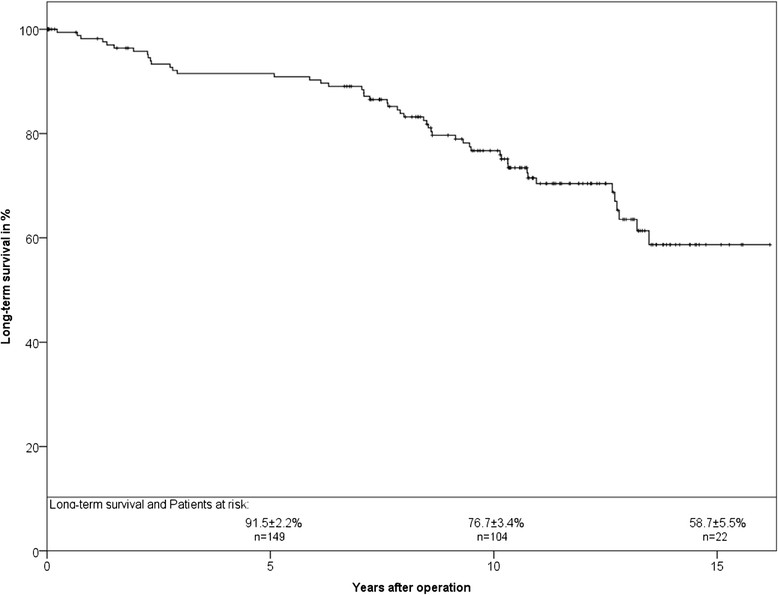
**Long-term survival of the study population in Kaplan-Meier analysis.**

Survival analysis of the whole study population is presented in Figure 1. At 5 and 10 years a long-term survival of 91.5 ± 2.2% and 76.7 ± 3.4%, respectively, was observed.

**Figure 2:**
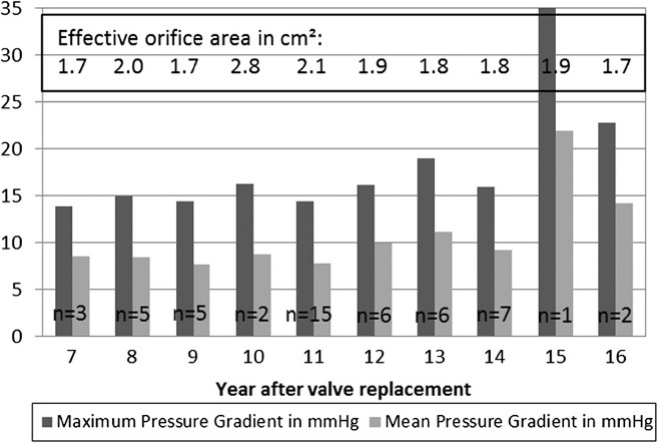
**Pressure gradient and effective orifice area depending on the year after implantation.**

**Figure 3:**
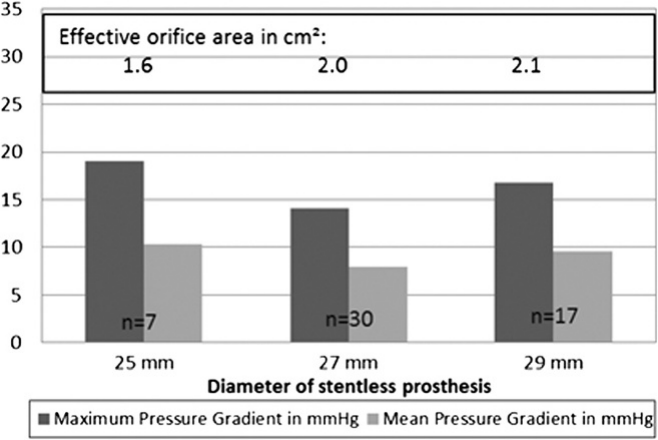
**Pressure gradient and effective orifice area depending on labelled size of valve.**

Maximum and mean pressure gradients were 16.3 ± 7.4 mmHg and 9.1 ± 4.2 mmHg, respectively. The mean pressure gradients of the prostheses at time of follow-up decreased 20.8% compared to pressure gradients directly after implantation of SAVR. Figure 2 shows maximum and mean pressure gradients depending on duration of implantation. No relevant difference could be found between the different intervals of implantation until 14 years after implantation. Afterwards one can see a rise in transvalvular gradients. Different sizes of the implanted SAVR did not lead to relevant differences between pressure gradients, but different EOAs could be observed (Figure 3).

**Figure 4:**
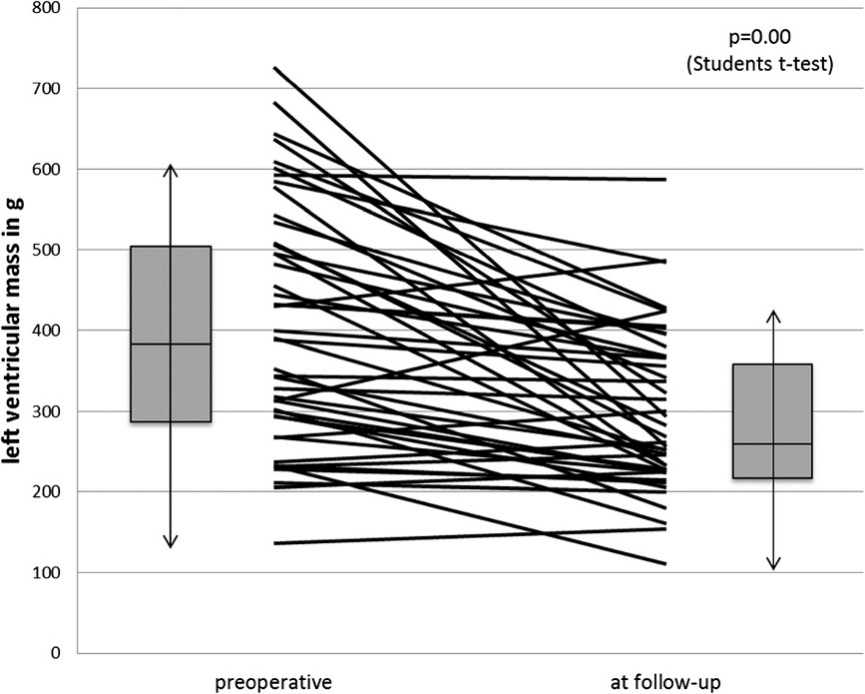
**Development of left ventricular mass in each patient (lines) and for the whole echocardiographicly examined study cohort (Boxplots: box equals interquartile range with median and whiskers of 1.5× interquartile range).**

Left ventricular mass was captured in all except 9 patients prior to the aortic valve replacement and in all patients at follow-up. Considering this, a reduction of left ventricular mass in 84% of the patients was achieved (Figure 4). Analysis of all examined patients showed a reduction in left ventricular mass of 26.5% (p < 0.01). For patients with preoperative hypertrophy of more than 170 g/m^2^ a reduction of 30% could be observed (p < 0.01). Between the different time intervals (7–16 years) after operation no significant differences were found, pointing to an already completed left ventricular remodeling 7 years after the operation.

**Figure 5:**
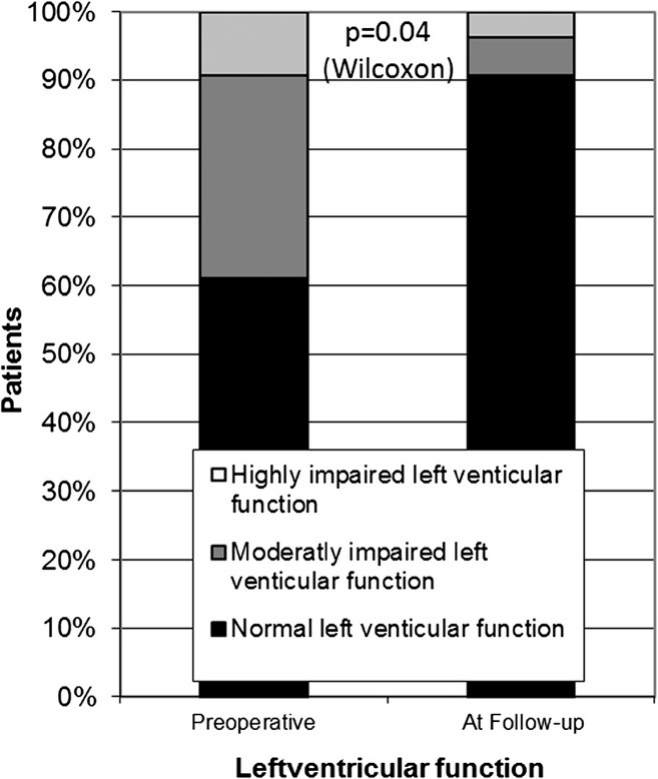
**Development of left ventricular function.**

Left ventricular function increased in 17 patients and decreased in one patient while in remaining patients it persisted (Figure 5). For the whole cohort an increase of left ventricular ejection fraction of 3.67 ± 1.7% could be seen (p = 0.03), for the patients with preoperative impaired left ventricular function an increase of 13.0 ± 3.0% could be observed (p < 0.01).

One patient showed a severe regurgitation. The prosthesis was implanted 15 years ago and 3 months after the follow-up a successful reoperation was performed. Additionally, we found 4 cases with moderate insufficiencies. These patients received the SAVR 13–14 years ago and showed no clinical symptoms of the aortic insufficiency. The remaining 49 patients showed no or trivial insufficiencies.

**Figure 6:**
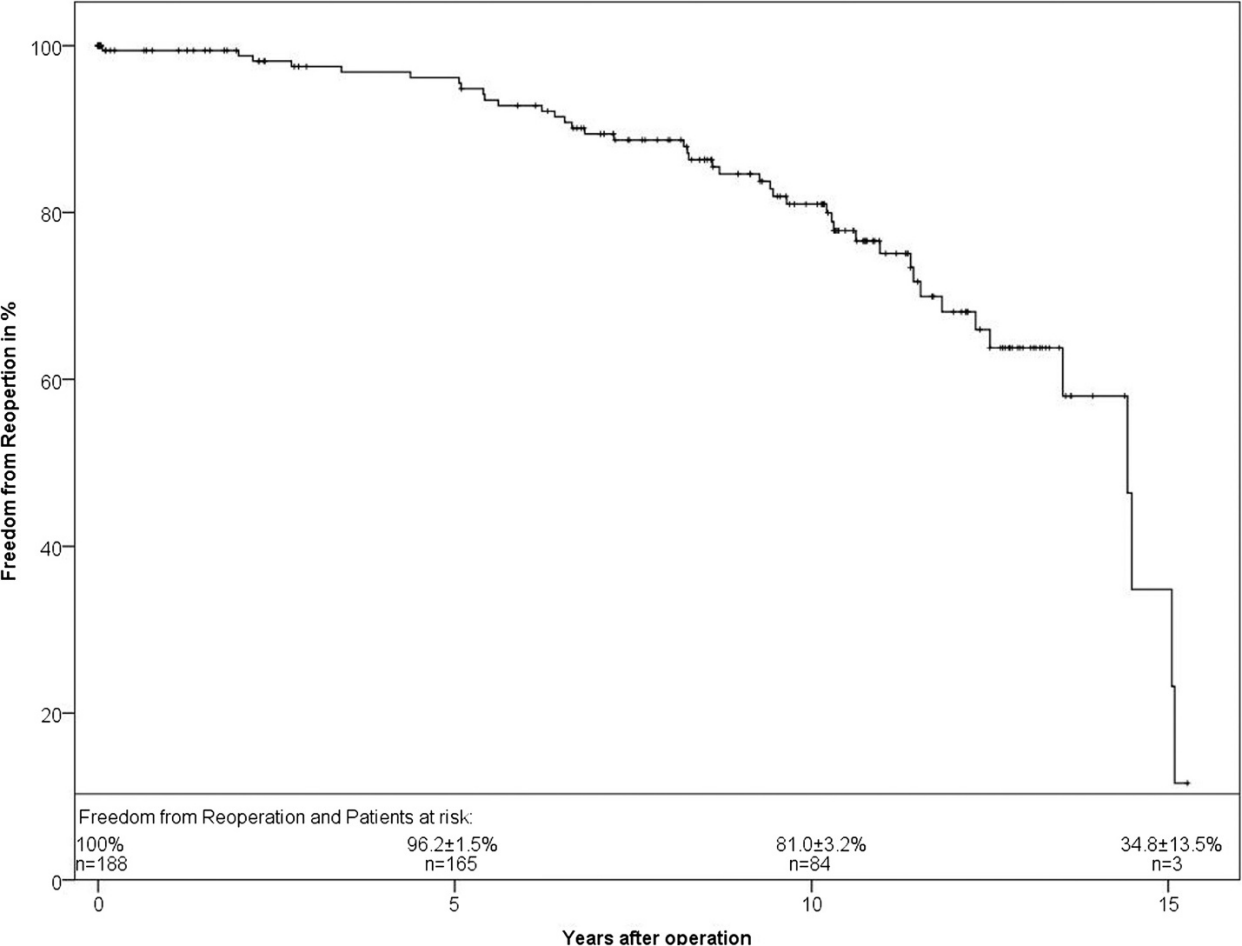
**Freedom from reoperation of the study population in Kaplan-Meier analysis.**

Freedom from reoperation at 5 and 10 years was 96.2 ± 1.5% and 81.0% ± 3.4%, respectively (Figure 6).

## Discussion

Stentless aortic valves promised excellent hemodynamic behavior mediated by a larger orifice area and a more physiological flow pattern. Short-term and mid-term follow-up, recapitulated in a meta-analysis by Kunadian [2], confirmed this excellent hemodynamic behavior in comparison to stented bioprostheses. The author showed that the use of a SAVR resulted in a larger effective orifice area and lower transvalvular gradients compared to stented valves. Our long term results showed constantly low transvalvular gradients up to 14 years after implantation. EOAs were calculated 1.6, 1.8 and 2.1 cm^2^ for 25, 27 and 29 mm valve sizes, respectively. However, captured pressure gradients were lower than for most stented bioprostheses, regardless of intraannular or supraannular implantation. For example, the Edwards Lifesciences Perimount Magna™ and Medtronic Hancock II® showed for the labeled sizes 25 mm, 27 mm, 29 mm EOAs of 1.4 cm^2^, 1.5 cm^2^, 1.8 cm^2^ and 1.3 cm^2^, 1.4 cm^2^, 1.8 cm^2^, respectively [12]. For the stented bioprosthesis St. Jude Medical Trifecta™ lower pressure gradients or higher EOA’s for the 3 different valve sizes have been reported [13]. This newer bioprosthesis, however, still has to stand the test of time.

The hemodynamic properties have an impact on the regression of left ventricular mass and left ventricular remodeling. Left ventricular hypertrophy develops in patients with aortic stenosis as an adaptation to increased pressure load. Incomplete regression of left ventricular hypertrophy after aortic valve replacement is associated with an increased mortality [14]. The observed regression of left ventricular hypertrophy in our study is a consequence of the hemodynamic properties of the SAVR. The remodeling process is also leading to an improvement of left ventricular function, which was observed in our study. Additionally, no relevant insufficiency could be shown till 14 years after operation. This lack of volume overload also supports the left ventricular remodeling.

The limited durability of biological valves and consecutive reoperations are the major concern for the use of bioprostheses in younger patients. Therefore current guidelines recommend the use of mechanical AVR in patients under 60 years [5]. The superior hemodynamic performance of SAVR could lead to a better durability due to less stress on the cusps of the SAVR. The previously published freedom from reoperation with a median of 14.43 ± 0.54 years in our cohort [7] supports this hypothesis. Literature research showed that most other biological aortic valves in younger patients tend to fail earlier [7],[8]. Our data showed an at least similar durability of SAVR compared to stented bioprostheses in younger patients for the first 14 years [7]. However, durability is inferior to mechanical valves. The risk of reoperation in case of bioprostheses must be weighed against the risk of thromboembolic or bleeding events with a mechanical valve [15]. Ruel et al. [6] showed that these different risks regarding valve replacements led to slightly superior survival in patients with bioprostheses during 25 years follow-up.

Aortic valve repair with various valve sparing techniques is an alternative to valve replacement and often used in younger patients. These procedures may be a useful option in carefully selected patients, mostly with isolated aortic valve regurgitation. Minakata et al. [16] published reoperation rates of 11% and 15% at 5 and 7 years, respectively. A systematic review by Carr et al. [17] reported 5 and 10 year freedom from reoperation of 89% and 64%. These data led to concerns about long term durability after aortic valve repair and are inferior to the presented results with stentless bioprostheses.

The impact of the hemodynamic performance of SAVR on patient-survival has been shown in midterm follow-up [4]. Figure 1 shows the long-term survival of our study population. Detailed characteristics of this study cohort were previously published [7]. For patients with isolated aortic valve stenosis and normal left ventricular function survival was comparable to the general population [7]. Additionally, comparison with the literature showed that mortality in our study population was comparable to mechanical or stented biological aortic valves [7],[8]. Thus, SAVR led to the same survival during long-term follow-up with better hemodynamic properties.

### Limitations

A limitation is the small study population, especially at 15 and 16 years after the operation. The implantation of SAVR in patients aged 60 years or younger, however, was not the standard procedure and no other long-term follow-up for such a cohort is available. Cardiac sonographers and echocardiography systems have changed over the 16 years of follow-up, which may lead to a bias of data.

## Conclusion

SAVR in patients aged 60 years or younger provides excellent hemodynamic long-term results without significant rise of transvalvular pressure gradients or profound regurgitation until 14 years after the operation. Consequently, reoperation rates remained low. The hemodynamic performance led to left ventricular mass regression and improvement of left ventricular function in the study population, particularly in patients with advanced myocardial hypertrophy or poor left ventricular function. Thus, SAVR is an alternative to mechanical valves or valve sparing techniques in younger patients.

## Authors’ original submitted files for images

Below are the links to the authors’ original submitted files for images. Authors’ original file for figure 1 Authors’ original file for figure 2 Authors’ original file for figure 3 Authors’ original file for figure 4 Authors’ original file for figure 5 Authors’ original file for figure 6
