# Supplementary figures and images for: Hemodynamic behavior of stentless aortic valves in long term follow-up
Source: J Cardiothorac Surg. 2014 Dec 20;9:197. doi: 10.1186/s13019-014-0197-2 (PMC4297443; doi:10.1186/s13019-014-0197-2)

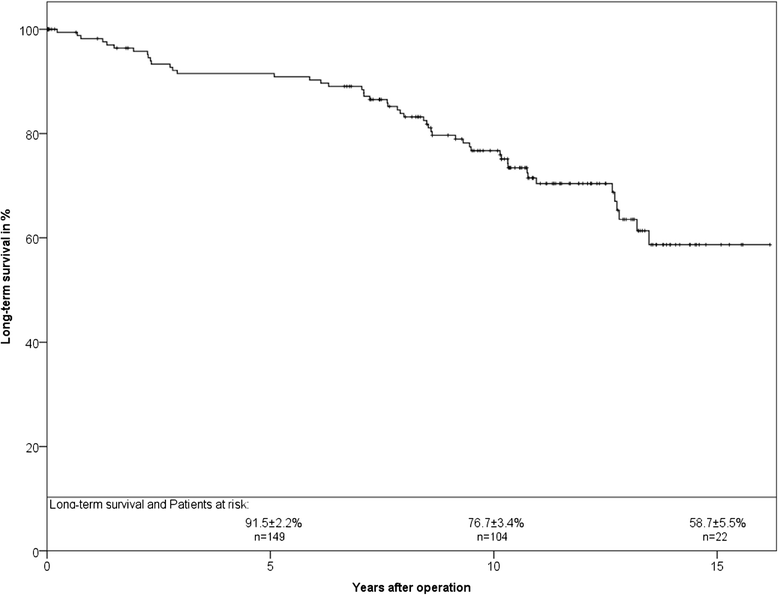

Supplement: Supplementary file 1 — Authors’ original file for figure 1 [file 13019_2014_197_MOESM1_ESM.gif]

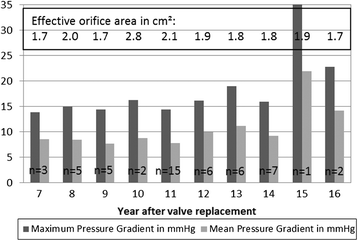

Supplement: Supplementary file 2 — Authors’ original file for figure 2 [file 13019_2014_197_MOESM2_ESM.gif]

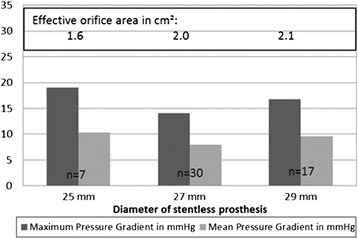

Supplement: Supplementary file 3 — Authors’ original file for figure 3 [file 13019_2014_197_MOESM3_ESM.gif]

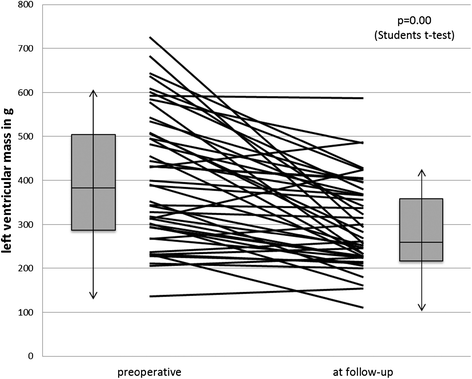

Supplement: Supplementary file 4 — Authors’ original file for figure 4 [file 13019_2014_197_MOESM4_ESM.gif]

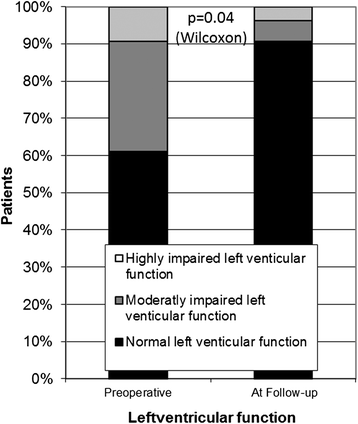

Supplement: Supplementary file 5 — Authors’ original file for figure 5 [file 13019_2014_197_MOESM5_ESM.gif]

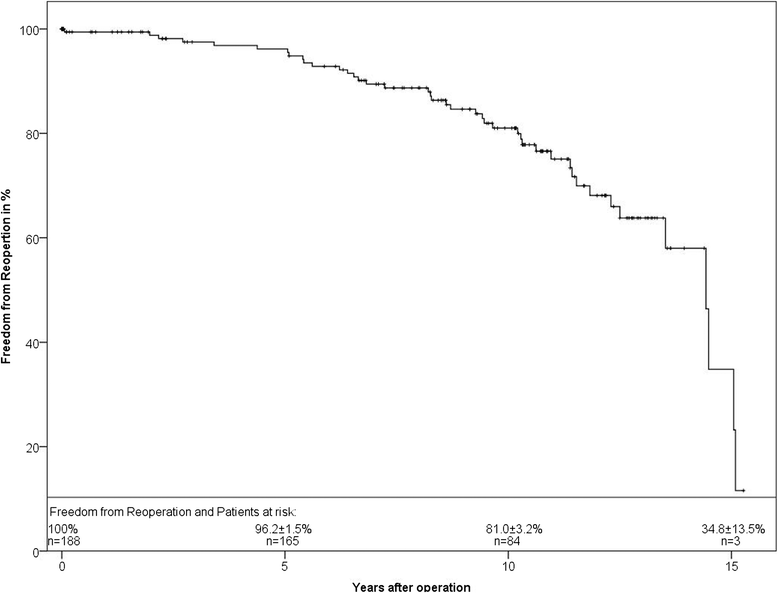

Supplement: Supplementary file 6 — Authors’ original file for figure 6 [file 13019_2014_197_MOESM6_ESM.gif]
